# Avoiding Surgery: Endoscopic Treatment of Congenital Duodenal Stenosis

**DOI:** 10.1097/PG9.0000000000000347

**Published:** 2023-08-16

**Authors:** Jessica V. Baran, Jerry M. Brown, Desiree Rivera-Nieves, Sara Karjoo, C. Jason Smithers, Michael J. Wilsey

**Affiliations:** From the * Office of Medical Education, Florida Atlantic University Charles E. Schmidt College of Medicine, Boca Raton, FL; † Department of Gastroenterology, Johns Hopkins All Children’s Hospital, Saint Petersburg, FL; ‡ Department of Surgery, Johns Hopkins All Children’s Hospital, Saint Petersburg, FL

**Keywords:** radial incisional therapy, endoscopic balloon dilation, intralesional corticosteroid injection, endoscopy, membranous duodenal web

## Abstract

Duodenal stenosis is a rare congenital anomaly that is typically treated surgically, although endoscopic incisional therapy (EIT) and balloon dilation are minimally invasive alternatives. We present a case of a 15-month-old male with vomiting and difficulty tolerating solid food due to severe congenital duodenal stenosis. The patient underwent EIT and serial duodenal dilation to a diameter of 20 mm, which resulted in significant symptom improvement. Intralesional corticosteroid injection (ISI) was administered to help prevent the duodenal septum from restricturing. The combination of EIT, balloon dilation, and ISI was successful in treating the patient’s congenital duodenal stenosis and avoided the need for surgery. However, further studies are required to confirm the efficacy of this treatment approach in this patient population. This report highlights the potential of this minimally invasive approach as an alternative to surgical intervention in the management of congenital duodenal stenosis.

## INTRODUCTION

Congenital duodenal anomalies occur with an estimated 1 in 20 000–40 000 births (1). Duodenal stenosis accounts for 2% of all noted congenital duodenal anomalies (1). This is caused by various intrinsic defects, resulting in a membrane-like stricture comprised of mucosa and submucosa that partially or completely obstructs the duodenum, with duodenal atresia representing the most severe presentation (2). Traditional therapies include open or laparoscopic duodenoduodenostomy or duodenojejunostomy (1). Surgical intervention increases the patient’s risk of surgical complications, including wound infection, anastomotic leakage, hepatobiliary injury, intestinal adhesions, and ventral hernias (1). With the recent advancement of endoscopic therapy, including balloon dilation and endoscopic incisional therapy (EIT), advanced endoscopists can offer endoluminal treatment for duodenal anomalies in children with reduced recovery time (2) while avoiding potential postsurgical complications (1–9).

A recent study by Ngo et al suggests that corticosteroid injection following balloon dilation in children with esophageal atresia anastomotic strictures improves stricture diameter more than dilation alone (10). The proposed mechanism suggests corticosteroids may limit the postdilation inflammatory response to help reduce collagen formation and scarring (10). Currently, there are no reports in the literature of corticosteroid use after endoscopic dilation of congenital duodenal anomalies. Herein, we describe the successful clinical application of concomitant EIT, balloon dilation, and intralesional corticosteroid injection (ISI) in a small child with congenital duodenal stenosis, avoiding the need for surgery.

## CASE REPORT

**FIGURE 1.**
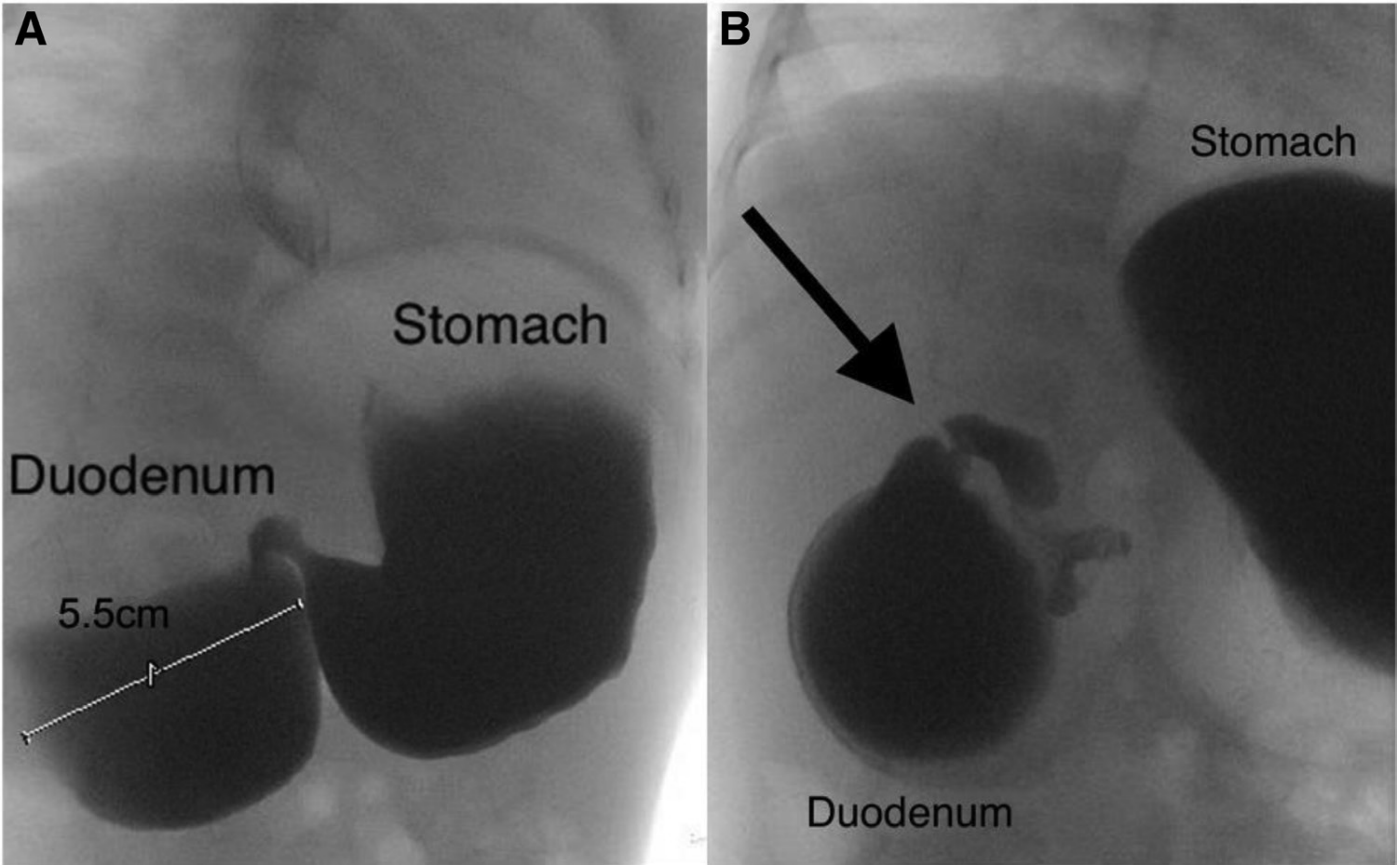
Fluoroscopic UGI series images showing: (A) “Double-bubble sign” with proximal duodenum dilated to 5.5 cm in diameter; and (B) high-grade duodenal obstruction associated with severe duodenal stenosis (arrow).

A 15-month-old full-term male with a history of vomiting beginning in infancy presents with recurrent vomiting 1–2 times/week, often post-prandial and worse with solid food intake. He was eating a primarily liquid diet with an inability to tolerate any solid food due to vomiting. He was treated with famotidine without improvement. An upper gastrointestinal series at an outside hospital revealed a “double bubble sign” (Fig. 1A), with severe duodenal stenosis between the descending and horizontal duodenum, distal to the Ampulla of Vater (Fig. 1B). The patient was transferred to Johns Hopkins All Children’s Hospital for endoscopic evaluation and further management.

**FIGURE 2.**
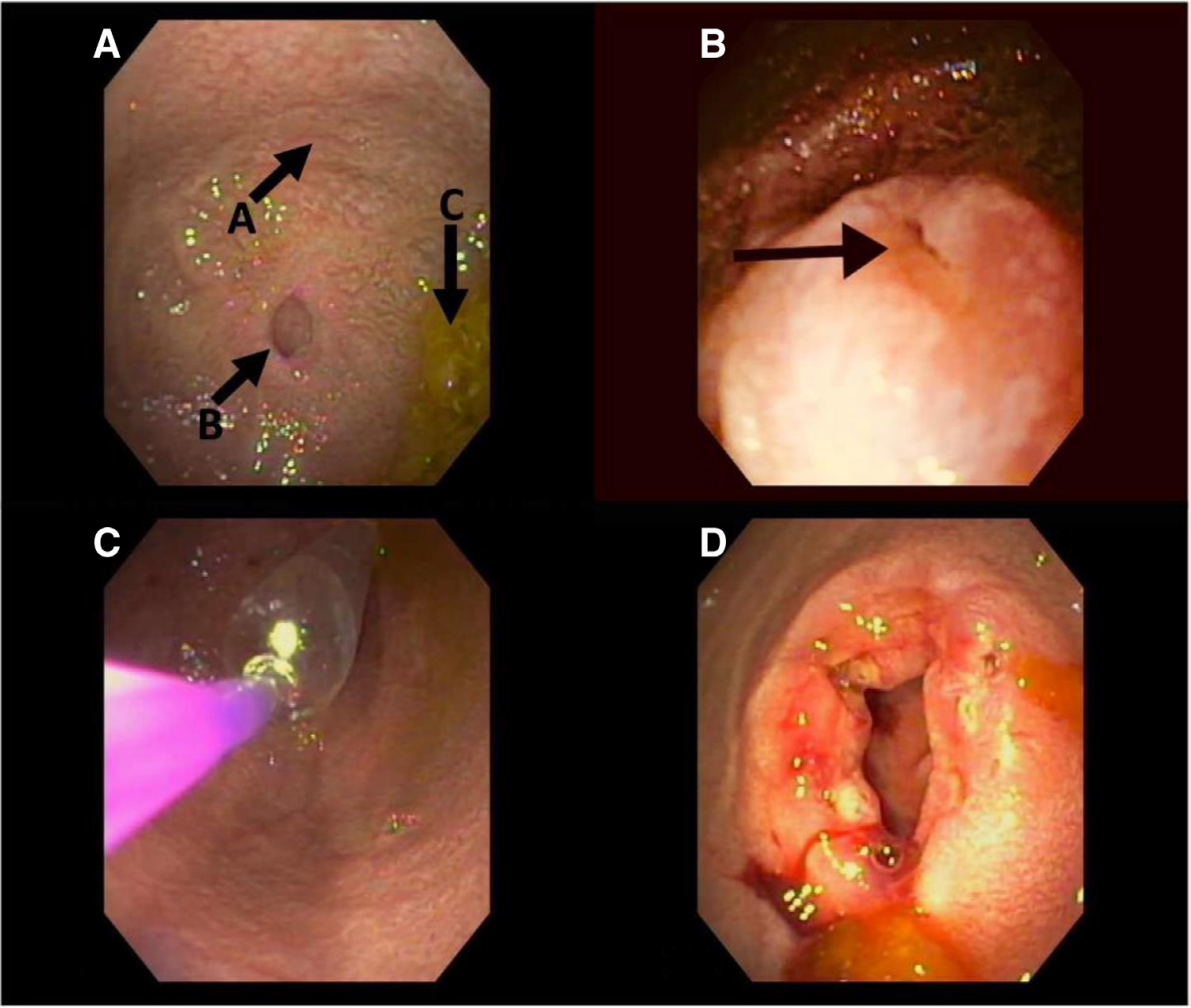
Significant duodenal dilation (A) noted proximal to the duodenal stenosis with pinhole orifice (B) just distal to the Ampulla of Vater on initial endoscopy. Bile was noted to pool (C) in the dilated duodenum. (B) Close-up view of pinhole orifice of the duodenal stenosis (arrow). (C) Endoscopic image showing balloon dilation of severe duodenal stenosis. (D) Patent duodenal orifice, status post endoscopic incisional therapy, and balloon dilation to 20 mm.

Upper endoscopy revealed marked proximal duodenal dilation (Fig. 2A). After fluid removal, a small pinhole orifice (<2 mm) was seen in the center of the congenital duodenal stenosis (Fig. 2B). A guidewire was inserted through the pinhole orifice and was noted in the small bowel by fluoroscopy. The 6–7–8 mm CRE Balloon Dilatation Catheter (Boston Scientific, Marlborough, MA) was inserted through the pinhole orifice and sequentially dilated to 6 to 7 to 8 mm. Next, the ITknife nano (Olympus Medical Co., Tokyo, Japan) needle knife was used to make the endoscopic incisions 3–4 mm deep at the 12, 4, and 8 o’clock positions. This allowed further dilation from 10 to 11 to 12 mm. No further dilation was performed due to minor bleeding that stopped spontaneously. The patient was discharged home that afternoon once tolerating a liquid diet, with a follow-up endoscopy and EIT scheduled 2 weeks later.

The patient’s symptoms improved following the first EIT and balloon dilation. His vomiting improved and he was eating pureed foods and soft solids for the first time. At the second dilation, 6 EIT incisions were made, and the stenosis was dilated to 18 mm (Fig. 2C). He was discharged home afterward. The case was discussed with our esophageal atresia surgeon (C.J.S.), who suggested that ISI may help decrease the postdilation inflammatory response. Therefore, a third dilation was scheduled 6 weeks later in which 5 EIT incisions were performed, and the stenosis was dilated to 20 mm (Fig. 2D). Additionally, 12 mg of triamcinolone (~1 mg/kg/dose) was injected in aliquots along the 4 quadrants circumferentially, implementing our hospital protocol for corticosteroid injection for esophageal strictures and applied it to duodenal stenosis. A telehealth visit 6 weeks later revealed the patient was now eating table food, the vomiting resolved, and his weight was up 2.9kg (current weight 14.5kg) since the last dilation. We recommended a follow-up upper gastrointestinal series (UGI series) in 6 months to evaluate the stenosis, but when the time came the parents declined the UGI series due to the resolution of vomiting and feeding intolerance, markedly improved growth and weight gain, and the family’s concerns regarding the ongoing COVID-19 pandemic. The family was subsequently lost to follow up.

## DISCUSSION

Duodenal stenosis is a rare congenital anomaly that typically presents early in life. Traditional surgical management includes open or laparoscopic duodenoduodenostomy or duodenojejunostomy (1), but these surgeries can be associated with intraoperative and postoperative surgical complications (1). Endoluminal therapy performed by advanced endoscopists may avoid many potential surgical complications and shorten procedure time and length of hospital stay. Our patient’s mean endoscopic procedure time was 39 minutes (Table 1). Our patient was discharged home after the first endoscopy with an immediate improvement in symptoms. Two subsequent endoscopies were performed on an outpatient basis, dilating the severe stenosis from a pinhole to 20 mm in diameter.

**TABLE 1.** Characteristics of endoscopic incisional therapy and balloon dilation procedures for a toddler with severe duodenal stenosis

	Dilation #1	Dilation #2	Dilation #3
Weight (kg) at time of procedure	10.4	11	11.6
Number of endoscopic incisions	3	6	5
Incision depth (mm)	3–4	3–4	3–4
Estimated initial duodenal stenosis diameter (mm)	<2	7–8	10
Initial balloon diameter (mm)	6	10	12
Final balloon diameter (mm)	12	18	20
Intralesional steroid given after dilation (y/n)	No	No	Yes
Procedure time (min)	38	43	37
Room time (min)	56	59	64
Intubated (y/n)	Yes	No	Yes
Outpatient procedure (y/n)	Yes	Yes	Yes
Same day discharge (y/n)	Yes	Yes	Yes

EIT = endoscopic incisional therapy.

Reports of endoluminal therapy for duodenal lesions in pediatric patients are growing and appear effective in the hands of experienced advanced endoscopists (2–9). Goring et al reported a 2-center case series of 15 pediatric patients (7). All patients underwent balloon dilation, and 12 of 15 received EIT, with 2 having a second duodenal web diagnosed (7). Endoluminal therapy successfully treated 60% of patients with 1 endoscopic procedure. Another 20% required 2–3 procedures, and 20% required surgery (7). We believe the addition of intralesional corticosteroid injection may help decrease postdilation inflammation and fibrosis to help achieve long-term luminal patency.

This case report demonstrates the clinically successful application of ISI therapy following EIT and balloon dilation for congenital duodenal stenosis. While it is recommended to obtain a repeat UGI series to confirm the technical success of the radial incisional therapy with balloon dilation and ISI, the patient was lost to follow up due to symptom resolution and parental concerns over the COVID-19 pandemic. Despite this limitation, this case further shows that serial endoscopic balloon dilation with EIT allows for the clinically successful treatment of duodenal stenosis under direct visualization, is associated with a short procedural time, avoids an invasive operation, promotes prompt recovery, and may be performed effectively in the outpatient setting with potential cost savings. Careful patient selection is critical in achieving successful endoscopic management in these patients. Factors such as the location, length, and tortuosity of the stricture, as well as the underlying etiology, can greatly impact the success of endoscopic treatment.

Two recent meta-analyses suggest ISI is a safe and effective treatment in young children with refractory esophageal strictures (11,12). We believe corticosteroid injection in combination with EIT and balloon dilation may lead to less postdilation inflammation, scarring, and perhaps improved outcomes for infants and children with congenital duodenal stenosis. Further studies are needed to confirm the efficacy of this treatment approach in this specific patient population.

## ACKNOWLEDGMENT

Informed consent was obtained from the parents to publish this work.
